# Healthcare system indicators associated with modern contraceptive use in Ghana, Kenya, and Nigeria: evidence from the Performance Monitoring and Accountability 2020 data

**DOI:** 10.1186/s12978-019-0816-4

**Published:** 2019-10-26

**Authors:** Ibitola Asaolu, Velia Leybas Nuño, Kacey Ernst, Douglas Taren, John Ehiri

**Affiliations:** 1 Department of Health Promotion Sciences, Mel and Enid Zuckerman College of Public Health, University of Arizona, Tucson, USA; 2 Department of Epidemiology and Biostatistics, Mel and Enid Zuckerman College of Public Health, University of Arizona, Tucson, USA

**Keywords:** Healthcare policy, Healthcare system, PMA2020, Contraceptive use, Contraception, Family Planning, Reproductive health, Ghana, Kenya, Nigeria

## Abstract

**Background:**

Public health literature is replete with evidence on individual and interpersonal indicators of modern contraceptive use. There is, however, limited knowledge regarding healthcare system indicators of modern contraceptive use. This study assessed how the healthcare system influences use of modern contraceptive among women in Ghana, Kenya, and two large population states in Nigeria.

**Methods:**

This study used data from Phase 1 of the Performance Monitoring and Accountability 2020. The analytical sample was limited to women with a need for contraception, defined as women of reproductive age (15 to 49 years) who wish to delay or limit childbirth. Therefore, this analysis consisted of 1066, 1285, and 1955 women from Nigeria, Ghana, and Kenya respectively. Indicators of healthcare assessed include user-fees, visit by health worker, type of health facility, multiple perinatal services, adolescent reproductive healthcare, density of healthcare workers, and regularity of contraceptive services. All analyses were conducted with SAS (9.4), with statistical significance set at *p* < 5%.

**Results:**

The prevalence of modern contraceptive was 22.7, 33.2, and 68.9% in Nigeria, Ghana, and Kenya respectively. The odds of modern contraceptive use were higher among Nigerian women who lived within areas that provide adolescent reproductive healthcare (OR = 2.05; 95% C.I. = 1.05—3.99) and Kenyan women residing in locales with polyclinic or hospitals (OR = 1.91; 1.27—2.88). Also, the odds of contraceptive use were higher among Kenyan women who lived in areas with user-fee for contraceptive services (OR = 1.40; 1.07–1.85), but lower among Ghanaian women residing in such areas (OR = 0.46; 0.23—0.92). Lastly, the odds of modern contraceptive use were higher among women visited by a health-worker visit among women in Ghana (OR = 1.63; 1.11—2.42) and Nigeria (OR = 2.97; 1.56—5.67) than those without a visit.

**Conclusion:**

This study found an association between country-specific indicators of healthcare and modern contraceptive use. Evidence from this study can inform policy makers, health workers, and healthcare organizations on specific healthcare factors to target in meeting the need for contraception in Ghana, Kenya, and Nigeria.

## Plain English summary

This study examined the association between indicators of a healthcare system and modern contraceptive use in Ghana, Kenya, and Nigeria using data from the Performance Monitoring and Accountability 2020 (PMA2020) survey. Indicators assessed include user-fees for contraceptive services, health worker visit, multiple perinatal services, type of health facility, density of health workers, and regularity of contraceptive service. Women, aged 15–49 years, who demonstrated a need for contraception were retained in this study; they include 1285 women in Ghana, 1955 women in Kenya, and 1066 women in Nigeria. The percentage of women who used modern contraceptives was 22.7% in Nigeria, 33.2% in Ghana, and 68.9% in Kenya. Women in Kenya had higher chances of using modern contraceptive if they resided in an area with polyclinics or hospitals. Likewise, women in Nigeria had higher chances of using modern contraceptives if they lived in an area where health facilities provided reproductive healthcare to adolescents. Also, the chance of using modern contraceptives was higher among women visited by a health-worker in Ghana and Nigeria. Lastly, among women who resided in a locale where health facilities charged user fees for contraceptive services, the chance of modern contraceptive use were higher among women in Kenya, but lower among women in Ghana. Evidence from this analysis can inform tailored interventions that promote the healthcare system’s role in improving modern contraceptive use.

## Background

Because of its numerous benefits in preventing maternal death, decreasing preterm birth and low birthweight infants, and reducing neonatal and infant mortality, modern contraceptive is an integral part of reproductive, maternal, and child health interventions [1]. Nonetheless, in comparison with other regions, modern contraceptive uptake remains low in sub-Saharan Africa. In 2015, the modern contraceptive prevalence was 23.6% in sub-Saharan Africa whereas it was 57% in South-East Asia and 73% in Northern Europe [2]. This trend reflects a high proportion of unmet need for contraception in sub-Saharan Africa compared to other regions; in 2015 the prevalence of unmet need for contraception among women (aged 15–49) in a union was 24.2% in sub-Saharan Africa, 12% in southeast Asia, and 7.3% in northern Europe. Furthermore, scientific evidence points to the higher odds of unmet need for contraception and limited contraceptive use among women who report an unintended pregnancy [3–7]. Therefore, to decrease unmet need for contraception in sub-Saharan Africa, experts must continue to examine factors that promote the use of modern contraceptives.

The literature is replete with evidence of individual- and interpersonal-level predictors of contraceptive use including wealth, education, women’s decision-making abilities, and spousal support and communication [8–14]. For example, a previous analysis of the Demographic and Health Surveys assessed the association between Nigerian women’s decision-making and modern contraceptive use. The study found that between 2003 and 2013, women in Nigeria involved in their own healthcare had higher odds of using modern contraceptives [8]. Also, women’s participation in large household purchases was linked with use of modern contraception [8]. Even with increased decision making, women might be inhibited from accessing and using contraceptives because of inadequacies in the healthcare system of many sub-Saharan African countries [14].

Therefore, it is crucial to examine the role of the healthcare system in modern contraceptive use among women in sub-Saharan Africa. Over the past twenty years, there have been increased global programs to promote reproductive health. Two recent examples include the International Conference on Population and Development (ICPD) and 2012 Family Planning Summit. At the 1994 ICPD in Cairo Egypt, 179 countries agreed on a global consensus of a 20-year plan, named *Program of Action*. The *Program of Action* aimed to bolster sustainable development by ensuring individual human rights and dignity, including universal access to sexual and reproductive healthcare [15, 16]. Although the *Program of Action* is attributed with reducing child mortality and increasing access to contraception, many gaps remained in its implementation across various world regions. These gaps, identified in the 2014 ICPD’s *Framework of Action*, center mainly on adolescent health and unmet need for contraception in low- and middle-income countries [10]. Consequently, low- and middle-income countries bear the brunt of unmet need for contraception and limited reproductive health services [17, 18]; about 214 million women and girls with unmet need for contraception reside in these countries [18]. These figures point to the need for more efforts in increasing contraceptive use among those who desire them.

Another global undertaking that closely aligns with the aims of ICPD is the Family Planning 2020 (FP2020) initiative, an outcome of the 2012 London Summit on Family Planning. The FP2020 is a multi-sectorial partnership with the goal of providing an additional 120 million women and girls in poor countries access to contraceptives by 2020 [19]. The FP2020 initiative plans to achieve this goal by integrating contraceptive services into a continuum of care for women and children, promoting universal access to voluntary contraceptive information and services, ensuring strong partnerships between stakeholders, and assuring development of national contraceptive delivery plans [19]. FP2020 particularly focuses on healthcare systems by fostering country-level support, ensuring safe spaces for women and girls to access contraceptives, training health workers, decreasing delay in access to contraceptives, and scaling up successful interventions [19]. FP2020 is currently implemented in 69 countries across Africa, Asia, and Central and South America. Since FP2020’s inception in 2012, governmental donors have contributed $1.27 billion towards reproductive health and 46 million additional women and girls now use a form of modern contraceptive [19]. Although there has been an increase in modern contraceptive use many countries, there remains upward trend in the prevalence of women with unmet need for contraception in Central and West Africa [19].

Bearing in mind these undertakings, how then do we engage with healthcare systems to promote reproductive health in sub-Saharan Africa? We must consider a paradigm shift from a siloed healthcare-system approach to a holistic approach [20]. This system comprises three parts: health service delivery, resources and support systems, and governance [21]. Within the comprehensive healthcare system are factors, including level of care, health financing systems, availability of transportation and medical supplies, capacity of healthcare providers, and referral services [21]. The healthcare system is pertinent to understanding the indicators of contraceptive uptake because women may be denied reproductive healthcare if there is a lack of competent staff, limited contraceptive commodities, high cost of service delivery, and sparse health centers located far from clients. Understanding how the healthcare system influences contraceptive use allows public health stakeholders to identify factors—beyond individual and interpersonal determinants—that may hinder modern contraceptive use.

Various indicators of healthcare—such as service delivery, health financing, and health workforce—have been shown to improve modern contraceptive use. Integrating contraceptive services into existing maternal and child health program increases the uptake of contraceptive services [22, 23]. For example, in a randomized control trial, Rwandan women were more likely to use modern contraceptives when it was integrated into postnatal immunization services than women who did not receive contraceptive services during postnatal immunization services [22]. In addition, low socioeconomic status is often cited as a deterrent to contraceptive uptake and/or continuation [11, 24]. Therefore, modern contraceptive use is boosted through the provision of free or subsidized services to women who cannot afford contraceptives. An examination of youth in Madagascar showed that a voucher program that delivered education, counseling, and free contraceptives increased adolescents’ use of modern contraceptives, especially long-acting reversible contraceptives (LARCs) [25]. Lastly, a trained health workforce is pertinent to promoting contraceptive use. For example, a qualitative study of reproductive health providers in Uganda noted that many family planning clinics could not administer implants and intrauterine devices because the clinic lacked staff with the technical skills to insert these contraceptives [26]. When these three components (i.e. service delivery, health financing, and health workforce) of healthcare system function are present, it is easier for women who desire contraception to be counseled and provided with their preferred modern contraceptive (s).

Thus, the aim of this study is to understand how the three building blocks of a healthcare system—health financing, service delivery, and health workforce—affect modern contraceptive prevalence among women in sub Saharan Africa, using Ghana, Kenya, and Nigeria as case study examples from West and East Africa. Specifically, these countries were chosen because they were the first few countries with data from the PMA2020 initiative.

## Methods

The study used data from the Performance Monitoring and Accountability 2020 (PMA2020) survey. PMA2020, funded by the Bill and Melinda Gates Foundation, is the technical hub of the performance, monitoring, and accountability of the global FP2020 Initiative. The PMA2020 is led globally by the Johns Hopkins University and implemented domestically in: 1) Ghana by the Kwame Nkrumah University of Science and Technology; 2) Kenya by the International Center for Reproductive Health; 3) Nigeria by the Center for Research, Evaluation Resources and Development, Obafemi Awolowo University [27]. Using in-country resident enumerators, the PMA2020 provides de-identified (i.e. data has no personal identifiers to protect participant’s privacy) and nationally representative information on family planning among women aged 15–49 years. In addition, PMA2020 collects data on water, sanitation, and hygiene (WASH) and indicators of healthcare delivery from pharmacies, clinics, and hospitals. This study used the first round of the PMA2020 surveys from Ghana, Kenya, and Nigeria. While Ghana and Kenya have nationally representative surveys, only information from Lagos and Kaduna states are reflected in the Nigerian PMA2020 data. The PMA2020 data is collected biannually for the first two years and annually subsequently and the survey’s sampling methods have been previously described [27]. Briefly, the PMA2020 uses a two-stage cluster design (i.e. sample of enumeration areas was drawn from the national population’s sampling to reflect two strata, urban/rural area and region/state, and clusters of households within each stratum) to collect individual, household, and service point delivery data from enumeration areas [27]. All PMA2020 data are publicly available upon request. This study was considered exempt from the University of Arizona Institutional Review Board because the PMA2020 is a de-identified secondary survey.

### Measures

This study used both the female respondent questionnaire (in the household survey) and service delivery points (SDP) data contained in the PMA2020 survey. The household survey has information on individual woman’s contraceptive use, parity, age, and other socio-demographic characteristics. The SDP survey, however, contained information on the type of health services within a woman’s enumeration area. The number of SDPs surveyed include 143 facilities in Ghana, 277 in Kenya, and 231 in Nigeria. To gather a comprehensive data on both individual and healthcare correlates of contraceptive use, we merged both female respondent questionnaire in the household and SDP surveys using a unique identifier describing the cluster or enumeration area.

Nine healthcare-system indicators were examined in this study. Multiple perinatal service was measured if a health facility offered contraceptive services in addition to antenatal care, delivery, or postnatal care. Otherwise, when the health facility provided only contraceptive services, it was assumed that such a facility lacked integrated services. Other variables describing healthcare delivery were included in this analysis, including type of health facility (hospitals, health centers, or community-based health planning and services), type of healthcare management (government, private, or other), density of professional healthcare providers (doctors, nurses and midwives, and pharmacist), routine fee for contraceptive services, advanced facility (i.e. hospitals, health centers, or clinics), and a health worker visit pertaining to contraception. Adolescent reproductive healthcare was measured if health facilities provided the following service(s) to unmarried adolescents: contraception counseling, prescription or referral for contraceptives, or contraceptives.

Unmet need for contraception is the discrepancy between a sexually-active woman’s reproductive goal and her contraceptive behavior [28]. This concept is calculated as the proportion of women of reproductive age either married or in a union who want to limit or delay childbearing, but who are not using any method of contraception [29]. Therefore, women who are unable to bear children, menopausal, not sexually active, and those with no unmet need for contraception (i.e. women who desired current pregnancy or postpartum amenorrheic women who desired last birth) were excluded from the analysis [30]. The final analytic sample was limited to women with met- and unmet-need contraception. The initial sample consisted of 3722, 3809, and 3350 women who completed the female respondent questionnaire in Ghana, Kenya, and Nigeria respectively.

### Statistical analysis

The association between the healthcare system and women’s use of modern contraception was assessed by running Chi-Square tests and univariate and multivariable logistic regression models. Three covariates—age, education, and marital status—were included in the multivariable regression model for variable describing a health-worker visit in order to tease apart the different impacts of individual and healthcare indicators of contraceptive use. Since the PMA2020 is nationally representative, the analysis was weighted to reflect the generalizability of the data. Statistical significance was set at *p* < 0.05. All analyses were conducted on SAS 9.4 (Cary, NC).

## Results

Socio-demographic characteristic of respondents, their contraceptive use, and indicators of the healthcare system are displayed in Table 1. The analytic sample of the PMA2020 survey was limited to 1285 women in Ghana, 1955 women in Kenya, and 1066 women in Nigeria. The final sample consisted predominantly of married women; 79.6% in Ghana, 83.5% in Kenya, and 60.8% in Nigeria. The average age of respondents was 26.5 years in Nigeria, 29.4 years in Ghana, and 30.6 years in Kenya. The proportion of women with at least a secondary school education was 42.8% in Kenya, 56.8% in Nigeria, and 56.9% in Ghana. The average number of children per woman was 2.4 in Nigeria, 2.9 in Ghana, and 3.1 in Kenya. A low proportion of women in Kenya (14.1%) of Kenya, Nigeria (19.1%), and Ghana (24.2%) had a health-worker visit pertaining to contraception. The prevalence of modern contraceptive use among these women was 22.7, 33.2, and 68.9% in Nigeria, Ghana, and Kenya respectively.

**Table 1:** Characteristics of Respondents

	GHANA	KENYA	NIGERIA
	n (%)	n (%)	n (%)
Age (Mean; SD)	29.4 (8.0)	30.6 (7.8)	26.5 (9.0)
Parity (Mean; SD)	2.9 (2.2)	3.1 (2.2)	2.4 (2.5)
Marital Status			
Married or Cohabiting	1041 (79.6)	1653 (83.5)	645 (60.8)
Other	244 (20.4)	302 (16.5)	421 (39.2)
Education			
No Education	380 (24.0)	75 (4.2)	222 (21.9)
Primary Education	250 (19.1)	1121 (53.0)	234 (21.3)
Secondary or higher education	655 (56.9)	759 (42.8)	610 (56.8)
Modern Contraceptive Use			
No	873 (66.8)	614 (31.1)	807 (77.3)
Yes	412 (33.2)	1341 (68.9)	259 (22.7)
Visited by health worker			
No	952 (75.8)	1646 (86.0)	841 (80.3)
Yes	333 (24.2)	309 (14.1)	225 (19.7)
Multiple Perinatal Services			
No	185 (16.1)	284 (18.7)	330 (33.0)
Yes	1100 (83.9)	1671 (81.3)	736 (67.0)
Facility Provides and Prescribes Contraceptives to Adolescents			
No	76 (6.3)	478 (21.0)	559 (54.7)
Yes	1209 (93.7)	1477 (79.0)	507 (45.3)
Facility has routine fees for contraceptive services			
No	164 (14.7)	1567 (79.2)	471 (37.3)
Yes	1121 (85.3)	388 (20.8)	595 (62.7)
Advanced Facility			
No	170 (14.9)	203 (10.8)	855 (80.2)
Yes	1115 (85.1)	1752 (89.2)	211 (19.8)
Facility Type			
Hospital/Polyclinic	411 (34.4)	289 (11.7)	164 (14.4)
Health Center/Health Clinic	457 (33.4)	562 (41.5)	526 (49.0)
Other-including CHPS^a^/Pharmacy	417 (32.2)	1104 (46.8)	314 (36.6)
Management			
Other including NGO/Faith-based	23 (0.9)	36 (1.1)	18 (1.8)
Government	1062 (81.8)	1603 (79.0)	669 (59.3)
Private	200 (17.3)	316 (19.9)	379 (38.9)
Number of Days contraceptive services provided			
Less than 7 days per week	809 (64.6)	1687 (84.2)	472 (51.4)
Everyday	476 (35.4)	268 (15.8)	495 (48.6)
Average Number of Healthcare Workers per facility			
Doctors	6.2	1.1	1.4
Nurses	47.9	8.5	6.6
Pharmacist	1.1	0.4	3.0
TOTAL	1285 (100%)	1955 (100%)	1066 (100%)

^a^ = *CHPS* Community-based Health planning and services

Over a third (34.4%) of health facilities in Ghana were hospitals or polyclinics compared to 11.7% in Kenya and 14.4% in Nigeria. Most of Ghanaian (81.8%) and Kenyan (79.0%) health facilities were run by government agencies as opposed to 59.3% in Nigeria. Across all countries, less than half of health facilities provided contraceptive services daily; 15.8% of facilities in Kenya, 35.4% in Ghana, and 48.6% in Nigeria delivered contraceptive services daily. A large proportion of health facilities delivered multiple perinatal services: 67% in Nigeria, 81.3% in Kenya, and 83.9% in Ghana. Also, health facilities in Ghana (93.7%) and Kenya (79.0%) had a higher prevalence of adolescent reproductive healthcare than those in Nigeria (45.3%). Over 4 in 5 of health facilities in Ghana (85.1%) and Kenya (89.2%) were designated as advanced health facilities compared to 19.8% of facilities in Nigeria.

The average number of nurses or midwives in a health facility ranged from 6.6 in Nigeria to 47.9 in Ghana. The mean distribution of doctors in a health facility varied from 1.1 in Kenya to 6.2 in Ghana. Pharmacists were the least represented type of health workers surveyed, with an average number of 0.4 pharmacist per health facility in Kenya to 3.0 pharmacists per health facility in Nigeria. Finally, over 3 in 5 of all health facilities in Ghana (85.3%) and Nigeria (62.7%) charged routine fees for contraceptive services while 1 in 5 (20.8%) of facilities in Kenya charged fees.

Bivariate analysis of the association between women’s use of modern contraception and health system indicators are presented in Table 2. Fee for contraceptive services was significantly associated with modern contraceptive use among women in Ghana (30.6% vs. 48.1%; *p* < 0.05) and Kenya (75.4% vs. 67.2%; *p* < 0.05). A health worker visit was associated with women’s use of modern contraceptives in Ghana (40.6% vs. 30.8%; *p* < 0.05) and Nigeria (46.9% vs. 16.7%; p < 0.05). Also, there was a significant relationship between a facility’s delivery of adolescent reproductive healthcare and modern contraceptive use in Nigeria (30.1% vs. 16.5%; p < 0.05). Lastly, fee-for-contraceptive services was significantly associated with modern contraceptive use among women in Ghana (30.6% vs. 48.1%; p < 0.05) and Kenya (75.4% vs. 67.2%; p < 0.05).

**Table 2:** Chi-Square test of association between contraceptive use and respondents’ characteristics

	Modern Contraceptive Use (%)		
	GHANA	KENYA	NIGERIA
Marital Status			
Married or Cohabiting	36.5	70.0	33.5
Other	32.3	68.7	5.9*
Highest level of Education Completed			
No Education	30.3	33.6	11.0
Primary Education	30.2	67.8	26.6
Secondary or higher education	35.4	73.7*	25.7*
Multiple Perinatal Service			
No	31.1	71.6	23.9
Yes	33.6	68.3	22.1
Facility Provides and Prescribes contraceptives to adolescents			
No	43.5	73.3	16.5
Yes	32.5	67.7	30.1*
Facility has routine fees for contraceptive services			
No	48.1	67.2	24.4
Yes	30.6*	75.4*	21.6
Visited by health worker			
No	30.8	68	16.7
Yes	40.6*	74.6	46.9*
Advanced Facility			
No	32.6	71.8	22.4
Yes	33.3	68.6	23.6
Contraception services provided			
Less than 7 days per week	35.3	68	20.3
Everyday	29.3	73.7	20.8
Facility Type			
Hospital/Polyclinic	33.0	79.3	22.2
Health Center/Health Clinic	29.5	69.9	22.3
Other-including CHPS/Pharmacy	37.2	65.5*	23.3
Management			
Other including NGO/Faith-based	34.8	65.2	24.3
Govt	33.2	64.5	21.3
Private	33.2	70.9	25.4

* represents *p* < 0.05

Multiple logistic regression models examining the relationship between service delivery indicators and modern contraceptive use are presented in Table 3. Ghanaian (OR = 1.63; 95% C.I. = 1.11–2.42) and Nigerian (OR = 2.97; 1.56–5.67) women who received a visit from a health-worker had higher odds of modern contraceptive use compared to women with no visit. In Nigeria, the odds of modern contraceptive use were higher among women who resided in an area with a health facility that provided adolescent reproductive healthcare (OR = 2.05; 1.05–3.99). In Kenya, the odds of modern contraceptive use were higher among women that resided in an area with a hospital or polyclinic (OR = 1.91; 1.27–2.88). Also, the odds of modern contraceptives use among women increased with a rise in number of nurses or midwives in Kenya health facilities (OR = 1.01; 1.00–1.01). Compared to women residing in areas with free services, in Ghana, women had lower odds of using modern contraception if they lived in areas with fee-for-contraceptive services (OR = 0.46; 0.23–0.92). On the contrary, the odds of modern contraceptive use were higher among Kenyan women residing in locales with fees-for-contraceptive services (OR = 1.40; 1.07–1.85). There was no significant association between fee-for-contraceptive services and modern contraceptive use in Nigeria (OR = 0.75; 0.36–1.57).

**Table 3:** Logistic regression models measuring the association between health-system variables and respondent’s use of modern contraceptives

	GHANA	KENYA	NIGERIA
	aOR (95% C.I)	aOR (95% C.I)	aOR (95% C.I)
Fees for service	**0.46 (0.23–0.92)**	**1.40 (1.07–1.85)**	0.75 (0.36–1.57)
Respondent visited by health worker	**1.63 (1.11–2.42)**	1.39 (0.99–1.93)	**2.97 (1.56–5.67)**
Advanced Facility	1.06 (0.56–2.01)	0.90 (0.63–1.28)	1.22 (0.56–2.67)
Facility provides multiple perinatal services	1.14 (0.61–2.14)	0.91 (0.67–1.23)	1.01 (0.42–2.44)
Facility provides or prescribes family planning services to adolescents	0.64 (0.28–1.44)	0.80 (0.61–1.05)	**2.05 (1.05–3.99)**
Management			
Government vs. Other	0.83 (0.59–1.16)	1.25 (0.68–2.29)	**1.51 (1.03–2.22)**
Private vs. Other	0.80 (0.45–1.37)	1.33 (0.71–2.52)	1.36 (0.64–2.86)
Facility			
Hospital/Polyclinic vs. Other	0.77 (0.44–1.34)	**1.91 (1.27–2.88)**	1.28 (0.41–3.40)
Health Center/Health Clinic vs. Other	0.72 (0.40–1.30)	1.19 (0.91–1.56)	0.87 (0.38–1.98)
Contraceptive services provided daily (Yes vs. No)	0.79 (0.48–1.31)	1.15 (0.86–1.53)	1.08 (0.47–2.46)
Number of Healthcare Provider per facility			
Doctor	1.00 (0.97–1.04)	1.03 (1.00–1.06)	1.06 (0.95–1.17)
Nurses	1.002 (1.000–1.004)	**1.006 (1.001–1.010)**	1.01 (0.97–1.05)
Pharmacists	0.996 (0.99–1.01)	1.07 (0.98–1.18)	1.23 (0.74–2.05)

Multiple logistic regression models controlled for the effects of age, marital status, education, and parity on the relationship between health-system determinants and modern contraceptive use

Bolded numbers represent statistically significant relationships at *p* < 0.05

## Discussion

This study assessed the association between specific elements of the healthcare system [20] and use of modern contraceptives among women in Ghana, Kenya, and Nigeria. Compared to previous studies that focused on individual or interpersonal indicators, this study adds to literature by exploring healthcare system predictors of modern contraceptive use. Our analyses showed that modern contraceptive use was associated with the following components of a healthcare system: health financing—facilities with routine fee for contraceptive services; health worker (density nurses and midwives and health worker visits); and service delivery model (type of healthcare facility and provision of adolescent reproductive healthcare). This discussion focuses on the significance of cost and health-worker visits in promoting access to and use of modern contraceptives.

The cost of purchasing contraceptives has been listed as a deterrent to modern contraceptive use among women, particularly intrauterine devices (IUDs) [31, 32]. This study found that Ghanaian women residing around health-facilities that required fees for contraceptive services had lower odds of using modern contraception. This association has also been reported in a similar study conducted in Ghana, which documented that high price of contraceptives limited women’s use of modern contraceptives. Specifically, a hike in the price of contraceptives led to 3% decrease in contraceptive use among women [33]. This barrier points to the importance of health financing mechanisms that ensure availability of contraceptives and the need to provide subsidized or free contraception. Prior to 2012, the government of Ghana did not include contraceptives provision into its National Health Insurance Scheme (NHIS) [34]. But the National Health Insurance Act of 2012 allowed for coverage of contraceptive services under Ghana’s NHIS [35]. This law, however, has not been implemented [35]. In addition, there is no evidence of appropriation of governmental funds to include family planning services under Ghana’s NHIS. Therefore, due to delayed policy implementation of family planning services, many Ghanaian women and families who desire contraceptives could remain with an unmet need for contraception.

Conversely, the cost of contraceptives does not always constitute a deterrent to contraceptive uptake. As shown in this study, Kenyan women residing in areas with facilities that require user-fees for contraception have higher odds of using modern contraceptives. While acknowledging that contraceptive prevalence rate of Kenya is greater than many other African countries [2], it is important to note that Kenya has several national structures supporting the use of modern contraceptives. One of these structures is the Voucher Program, a subsidized fee-for-service health financing program that provides safe motherhood and family planning programs to women from poor households [36]. Women with these vouchers can approach healthcare providers for reproductive health services at no extra cost [36]. An evaluation of the voucher program showed the program was associated with increased number of family planning service visits at designated facilities [36]. Also, Kenyan women might have higher odds of using modern contraceptives because the country has provision specifically for reproductive health financing. As of 2009/2010, Kenya’s total spending on reproductive health was 13.8% of the total health expenditure, and in 2010, private health insurance controlled 12.5% of total reproductive health expenditures [37]. As exemplified in Kenya, subsidized health services and purposeful reproductive health financing can increase women’s uptake of modern contraception.

Lastly, in Nigeria, donor agencies serve as the main source of funding for family planning, with supplemental funding from the federal government [38]. Nonetheless, the burden of delivering family planning services in Nigeria falls on individual state government since the federal government does not cover the administrative cost of contraceptive services [39]. Thus, delivery and uptake of contraceptive services may vary across states because of the diverse capacity of each state to train and pay health workers to prescribe and administer contraceptives, cover the cost of transporting contraceptives to primary health care centers, and provide infrastructure to store these contraceptives [38].

In addition, this study reveals that women in Ghana and Nigeria who had a health-worker visit pertaining to contraception had higher odds of using modern contraceptives. While the PMA2020 does not specify the type of health worker who conducted the visit, data from the current SDP data showed that 42% of facilities in Ghana, 45% in Nigeria, and 55% in Kenya support CHW. Moreover, community health workers are often the health workers charged with home visits and care in low- and middle-income countries [40]. Therefore, in absence of the health worker specificity, this study will examine community health worker (CHW)s’ role in reproductive healthcare. CHWs are trusted community members who are trained outside of the formal nursing or medical curricula to provide various “health, promotional, and mobilization services” within their community [40]. In areas with sparse health facilities and skilled professionals, community health workers serve as bridges between individuals and healthcare services and are pivotal to successful maternal and child health programs.

In Nigeria, CHWs are integrated into the country’s health system providing primary healthcare [41]. In 2012, the scope of work for CHWs in Nigeria was expanded to meet the demand for health providers who can provide long-acting reversible contraceptives (LARC) such as implants and IUDs [40]. The broader scope of CHW practice allows for increased uptake of LARCs especially in communities that do not have access to advanced health facilities. However, the progress in CHWs’ delivery of LARCs has been stalled because of the limited number of trained CHWs who can insert and remove IUDs and implants [39, 42]. In Ghana and Kenya, however, CHWs are an informal part of the national health system even though they provide reproductive, maternal, and child health services [43, 44]. Although there is evidence for CHWs providing healthcare in Ghana, Kenya, and Nigeria, more effort is needed in integrating CHWs into national health systems, providing them with on-going educational opportunities in contraceptive counselling, and training them on delivering contraceptives including LARCs. These efforts can promote contraceptive uptake among women by enhancing patient knowledge, reducing or eliminating distance as a barrier to contraceptive use, and assuring the competency of health workers in delivering various contraceptive commodities and methods.

### Limitations

There are three limitations to this study. First, although the PMA2020 service delivery and female respondent surveys were collected from the same enumeration area, it is uncertain whether women received contraceptive services in their residential enumeration area. Nonetheless, this fact is unlikely to alter the association between indicators of healthcare and women’s use of modern contraceptives presented in this study. Second, the PMA2020 does not provide information on women’s health insurance status. Thus, potential confounders, such as health insurance status influencing the association between service delivery and modern contraceptives uptake, were not explored in this study. Third, studies have shown that long distance to health facilities constitute a deterrent to the uptake of contraceptives [45]. Unfortunately, PMA2020 lacks information on distance to health facilities. Therefore, the study could not explore distance to health facility as a barrier to contraceptive use among respondents.

### Implications for policy practice and future research

By incorporating service delivery indicators into reproductive health surveillance, PMA2020 has paved the way for understanding the interplay between individual health and healthcare system. Utilizing results from PMA2020’s surveillance and analysis, researchers should partner with client advocates, healthcare providers, healthcare organizations, and ministries of health in creating an evidence-based reproductive healthcare practice. An example of such practice is expanding the scope of CHW practice as is evident in Nigeria [39, 42]. As observed in this study, women who receive a visit from a health worker (most likely CHWs) had higher odds of modern contraceptive use. Therefore, in countries—such as Kenya and Ghana—where CHWs are not officially incorporated into the healthcare workforce, more efforts should be devoted towards integrating them into the health workforce and training them to provide counseling and delivery of contraceptives to women and couples especially those who live in rural and hard-to-reach settings. Also, this study shows unique associations between user-fees and modern contraceptive use among countries studied, thus revealing the peculiarity of each country’s demand for and expenditure on reproductive health services. Consequently, there is a need for further research on how various health finance mechanisms influence contraceptive use in sub-Saharan Africa.

## Conclusion

This study examined how components of the healthcare system can influence modern contraceptive uptake. In addition to ratifying global reproductive-health policies, it is important to adopt a healthcare system approach in promoting modern contraceptive use. Sub-Saharan African countries can boost their modern contraceptive prevalence rate through targeted governmental policies and resource allocation for contraception, broadening the scope of community health workers to provide contraceptive services including LARCs, and providing free or subsidized contraceptive services.

## Data Availability

The dataset used for this study are publicly available in the PMA2020 repository, [https://www.pma2020.org/].

## Abbreviations

CHW: Community Health Worker
FP2020: Family Planning 2020
ICPD: International Conference on Population and Development
IUD: Intrauterine device
LARCs: Long-acting reversible contraceptives
NHIS: National Health Insurance Scheme
PMA2020: Performance Monitoring and Accountability 2020
WASH: Water, sanitation, and hygiene
